# Vibegron for the Treatment of Patients with Dry and Wet Overactive Bladder: A Subgroup Analysis from the EMPOWUR Trial

**DOI:** 10.1155/2022/6475014

**Published:** 2022-04-13

**Authors:** David Staskin, Jeffrey Frankel, Susann Varano, Michael Kennelly, Diane K. Newman, Matt T. Rosenberg, Denise D. Shortino, Rachael A. Jankowich, Paul N. Mudd Jr

**Affiliations:** ^1^Tufts University School of Medicine, Boston, MA, USA; ^2^Seattle Urology Research Center, Seattle, WA, USA; ^3^Clinical Research Consulting, Milford, CT, USA; ^4^Carolinas Medical Center, Charlotte, NC, USA; ^5^Perelman School of Medicine, University of Pennsylvania, Philadelphia, PA, USA; ^6^Mid-Michigan Health Centers, Jackson, MI, USA; ^7^Urovant Sciences, Irvine, CA, USA

## Abstract

**Background:**

Overactive bladder (OAB) is characterized by urgency and frequency with (OAB wet) or without (OAB dry) urge urinary incontinence (UUI). In the phase 3 EMPOWUR trial, vibegron—a selective *β*_3_-adrenergic receptor agonist for the treatment of OAB—significantly improved daily number of urgency episodes and micturitions vs. placebo (*P* < 0.01). These post hoc analyses aimed to compare the efficacy of vibegron vs. placebo in OAB dry and wet populations.

**Methods:**

Patients were randomly assigned 5:5:4 to receive once-daily vibegron 75 mg, placebo, or tolterodine 4 mg extended release, respectively, for 12 weeks. Baseline criteria for OAB dry included an average of ≥8 micturitions, ≥3 urgency episodes, and <1 UUI episode per diary day and for OAB wet included an average of ≥8 micturitions and ≥1 UUI episode per diary day. Change from baseline in mean daily number of urgency episodes and micturitions was assessed in both populations.

**Results:**

Of the 1463 patients included in the full analysis set, 336 (23%) had OAB dry (vibegron, *N* = 123; placebo, *N* = 115; and tolterodine, *N* = 98), and 1127 (77%) had OAB wet (vibegron, *N* = 403; placebo, *N* = 405; and tolterodine, *N* = 319). Vibegron was associated with significant reductions (95% CIs of the least squares mean differences [LSMD] does not include 0) from baseline at week 12 vs. placebo in mean daily urgency episodes for the dry (LSMD [95% CI], ‒1.0 [‒2.0, ‒0.1]) and wet (‒0.6 [‒1.0, ‒0.1]) populations. Vibegron was associated with significant reductions from baseline at week 12 vs. placebo in mean daily micturitions for the dry (LSMD [95% CI], ‒0.8 [‒1.5, ‒ 0.1]) and wet (‒0.5 [‒0.8, ‒0.1]) populations. There were no significant differences in either outcome between tolterodine and placebo for either the dry or wet populations in this study.

**Conclusions:**

In this subgroup analysis from the EMPOWUR trial, vibegron was associated with significant reductions compared with placebo in urgency episodes and micturitions in both the OAB dry and wet populations, suggesting that vibegron is similarly efficacious for these endpoints in patients with and without UUI. This trial is registered with NCT03492281.

## 1. Introduction

Overactive bladder (OAB) is a clinical syndrome characterized by urgency and frequency, with or without urge urinary incontinence (UUI), referred to as OAB wet and OAB dry, respectively [1, 2]. OAB is highly prevalent among US adults ≥40 years old [3], and approximately two-thirds of patients are affected by OAB dry [2, 4]. Among women, the overall prevalence of OAB dry and OAB wet is similar (7.6% and 9.3%, respectively); however, the prevalence of OAB dry is higher than OAB wet among men (13.4% vs. 2.6%, respectively) [4]. Nevertheless, the strong and sudden urge to urinate, without or with UUI, is a cornerstone symptom that patients with OAB experience [1, 2]. Consequently, frequent urgency episodes (defined by the International Urogynecological Association and the International Continence Society as a complaint of a sudden, compelling desire to pass urine, which is difficult to defer) can significantly affect a patient's daily functioning and productivity, with more than half of men and women (65% and 67%, respectively) reporting that their OAB symptoms impact their daily lives [2, 5]. Urinary frequency and urgency are reported more often than UUI (85% and 54% vs. 36%, respectively) [2], and urgency has been shown to be the greatest predictor of patient bother associated with OAB in both men and women [6]. Furthermore, significant reductions in health-related quality of life and increases in symptoms of depression were reported among patients with OAB experiencing urgency compared with those not experiencing urgency [7].

The standard of care for OAB is behavioral therapy with or without the use of pharmacologic treatment as a first-line treatment option [8, 9]. Anticholinergics and *β*_3_-adrenergic receptor agonists are are recommended as a second-line treatment for OAB [8, 9]; however, use of anticholinergics is associated with adverse effects such as dry mouth and constipation [10], as well as an increased risk of falls [11], cognitive impairment [12], and dementia [13]. *β*_3_-adrenergic receptor agonists are available as pharmacologic treatments for OAB and are not associated with anticholinergic-related side effects such as dry mouth and cognitive impairment [10, 14, 15].

Although OAB dry affects a larger proportion of the OAB population, studies of pharmacologic treatment typically report results in the overall OAB population or in patients with OAB wet and commonly focus on UUI as the most bothersome symptom of OAB. As a result, treatment guidelines do not differentiate between patients with OAB dry or OAB wet and often neglect to address the need to manage the disruptive and core symptom of urgency associated with OAB [1]. However, one observational study has suggested that OAB wet was associated with improved outcomes after receiving solifenacin [16]. A separate observational study indicated that the presence of OAB wet predicts better efficacy outcomes with onabotulinumtoxinA [17], a third-line treatment option. Because most randomized controlled trials enroll patients with OAB irrespective of subtype or only enroll patients with OAB wet, there is limited evidence demonstrating that the available treatment options are efficacious in patients with OAB dry and effectively manage urinary urgency leading to meaningful improvements for patients.

Vibegron is a selective *β*_3_-adrenergic receptor agonist recently approved for the treatment of adult patients with OAB [18]. Vibegron acts by mediating relaxation of the detrusor muscle by selectively stimulating *β*_3_-adrenergic receptors, improving storage capacity without impeding voiding of the bladder [19, 20]. In the phase 3 EMPOWUR trial, once-daily vibegron 75 mg for 12 weeks showed statistically significant improvements compared with placebo in mean daily number of micturitions and UUI episodes (coprimary endpoints; *P* < 0.001, each) and in urgency episodes (key secondary endpoint; *P* < 0.01) [21]. It remains unknown if vibegron is equally efficacious in improving bothersome symptoms of OAB, such as frequency of micturitions and urinary urgency episodes, among patients with OAB dry vs. OAB wet. Here we present post hoc subgroup analyses from the EMPOWUR trial, comparing the efficacy of vibegron vs. placebo in the OAB dry and wet populations.

## 2. Methods

### 2.1. Study Design and Participants

Detailed methods of the full study design of the phase 3, randomized, double-blind, placebo- and active-controlled EMPOWUR trial (https://clinicaltrials.gov/ct2/show/NCT03492281) have been previously published [21]. In brief, adult patients (up to 15% of whom could be male) were enrolled if they had a history of OAB (defined as urgency, without or with UUI, usually associated with frequency and nocturia) ≥3 months prior to the screening visit. Up to 25% of patients in the EMPOWUR trial could have OAB dry, which was defined as an average of ≥8 micturitions, ≥3 urgency episodes, and <1 UUI episode per diary day based on a 7-day patient voiding diary at baseline. Criteria for OAB wet included an average of ≥8 micturitions and ≥1 UUI episode per diary day at baseline. Patients were randomly assigned 5:5:4 to receive once-daily vibegron 75 mg, placebo, or tolterodine 4 mg extended release (active control), respectively, for 12 weeks. Patients completed a voiding diary for 7 days before baseline and weeks 2, 4, 8, and 12.

The EMPOWUR trial was conducted in compliance with the International Council for Harmonisation of Technical Requirements for Registration of Pharmaceuticals for Human Use Good Clinical Practice. Investigators received institutional review board, research ethics board, or independent ethics committee approval prior to the initiation of the study. All patients provided written informed consent.

### 2.2. Assessments

The coprimary efficacy endpoints of the EMPOWUR trial were change from baseline at week 12 in mean daily number of micturitions and UUI episodes. UUI episodes were not assessed in this current analysis as patients with OAB dry by definition had <1 UUI episode per day at baseline. Key secondary efficacy measures included change from baseline in mean daily number of urgency episodes at week 12 and percentage of patients achieving ≥50% reduction from baseline in urgency episodes per day at week 12.

### 2.3. Statistical Analyses

Outcomes were assessed in the full analysis set (FAS), which included all randomized patients who received ≥1 dose of double-blind study drug and had ≥1 evaluable change from baseline micturition measurement. The FAS for the dry and wet populations was defined as all patients in the FAS meeting the definition for OAB dry or OAB wet, respectively, at study entry. A mixed model for repeated measures (MMRM) with restricted maximum likelihood estimation was used to analyze changes from baseline at weeks 2, 4, 8, and 12 in efficacy outcomes. The covariates included in the MMRM were study visit, baseline number of urgency episodes/micturitions, and treatment by study visit interaction. Statistical significance was determined if the 95% CI of least squares mean difference (LSMD) did not include zero. The formal analysis of efficacy outcomes compared vibegron with placebo; all other efficacy outcomes including comparisons between tolterodine and placebo were considered supportive.

Among patients with OAB dry, the percentage considered responders for the urgency episodes endpoint (i.e., achieving ≥50% reduction from baseline in the mean number of daily urgency episodes) was analyzed by an additional post hoc analysis using Cochran–Mantel–Haenszel risk differences, stratified by sex (female vs. male), with weights proposed by Greenland and Robins [22]. Multiple imputation methods were used to estimate missing values for the responder endpoint of ≥50% reduction in urgency.

## 3. Results

### 3.1. Patients

Of the 1463 patients included in the overall FAS, 336 (23%) had OAB dry (vibegron, *N* = 123; placebo, *N* = 115; and active control, *N* = 98), and 1127 (77%) had OAB wet (vibegron, *N* = 403; placebo, *N* = 405; and active control, *N* = 319; Supplementary Figure 1). Overall, the mean age was 60.2 years, and most patients were white (78.3%) and enrolled in the US (89.6%; Table 1). The demographics of patients with OAB dry and wet were, in general, similar to those in the FAS. However, a higher percentage of patients with OAB dry vs. the overall OAB population were men (29.5% vs. 14.8%, respectively) and were from the US (99.1% vs. 89.6%, respectively). Correspondingly, a higher percentage of patients with OAB wet vs. OAB dry were women (89.5% vs. 70.5%, respectively). At baseline, the mean daily number of urgency episodes and micturitions were comparable between the FAS and OAB dry and wet populations.

### 3.2. Efficacy Endpoints

#### 3.2.1. Mean Daily Urgency Episodes

**Figure 1:**
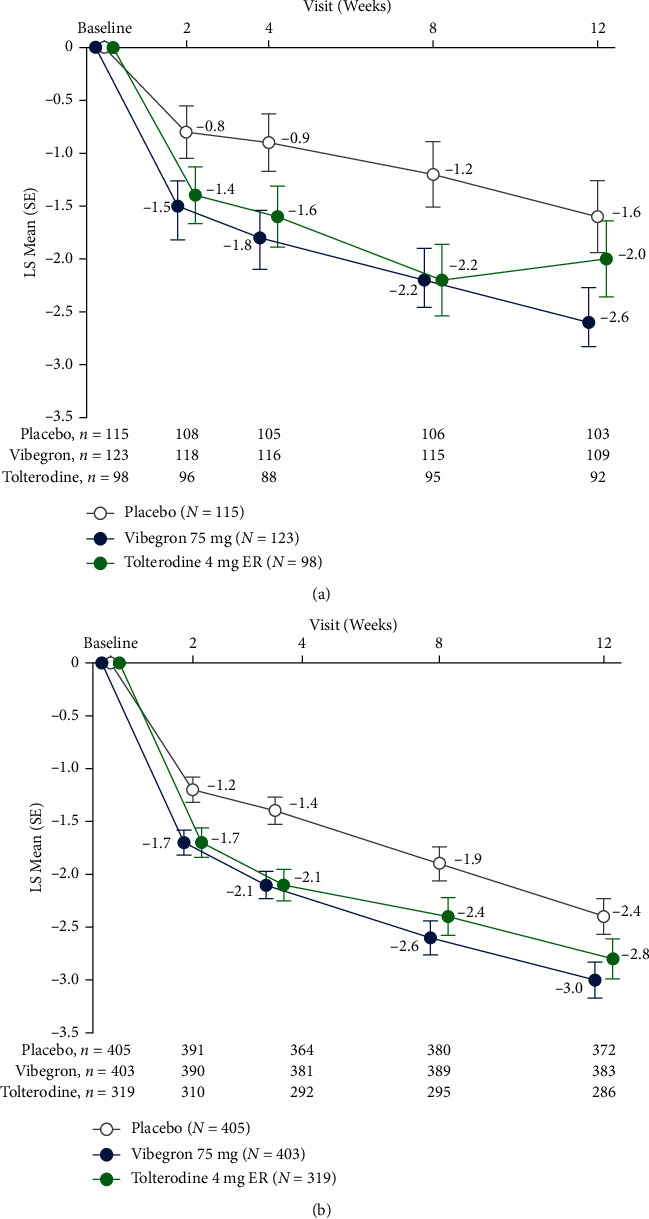
LS mean (SE) change from baseline in mean daily number of urgency episodes for patients with (a) OAB dry and (b) OAB wet. ER, extended release; LS, least squares.

Vibegron was associated with statistically significant reductions (95% CIs of LSMDs do not include 0) vs. placebo from baseline at week 12 in LS mean daily number of urgency episodes among patients with OAB dry (LSMD [95% CI], –1.0 [‒2.0 to ‒0.1]) and OAB wet (–0.6 [‒1.0 to ‒0.1]). The LS mean (95% CI) change from baseline at week 12 in mean daily number of urgency episodes for the OAB dry population was –2.6 (‒3.2 to ‒2.0) with vibegron vs. –1.6 (‒2.2 to ‒0.9) with placebo and for the OAB wet population was –3.0 (‒3.3 to ‒2.6) vs. –2.4 (‒2.7 to ‒2.0), respectively (Table 2). In both populations, significant (95% CIs of LSMDs do not include 0) reductions from baseline were also seen with vibegron vs. placebo beginning at week 2 and were maintained at weeks 4 and 8 (Figure 1; Supplementary Table 1). No significant differences in reductions (95% CIs of LSMDs include 0) from baseline at week 12 in the mean daily number of urgency episodes were seen between tolterodine and placebo for the dry or wet populations in this study.

#### 3.2.2. Mean Daily Micturitions

**Figure 2:**
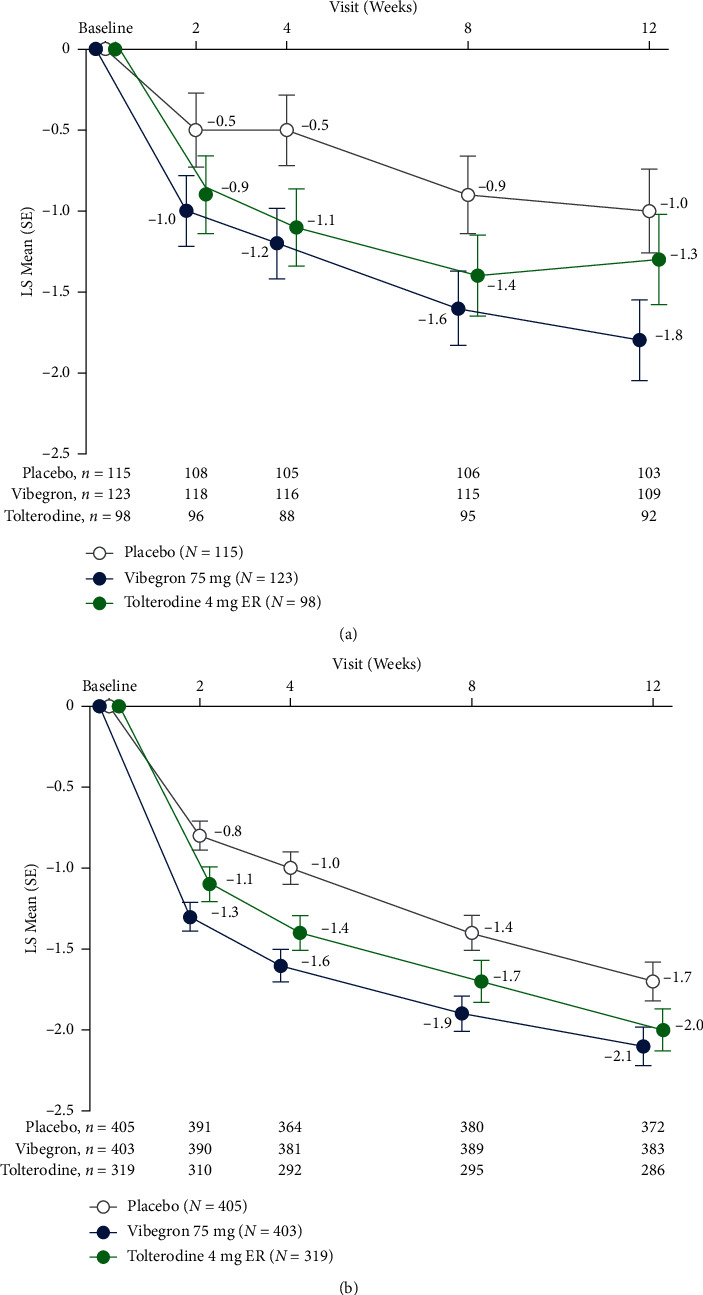
LS mean (SE) change from baseline in mean daily number of micturitions for patients with (a) OAB dry and (b) OAB wet. ER, extended release; LS, least squares.

Vibegron was associated with statistically significant reductions (95% CIs of LSMDs do not include 0) vs. placebo from baseline at week 12 in LS mean daily number of micturitions among patients with OAB dry (LSMD [95% CI], –0.8 [‒1.5 to ‒0.1]) and OAB wet (LSMD of –0.5 [‒0.8 to ‒0.1]). The LS mean (95% CI) change from baseline at week 12 in mean daily number of micturitions for the OAB dry population was –1.8 (‒2.3 to ‒1.3) with vibegron vs. –1.0 (‒1.5 to ‒0.5) with placebo and for the OAB wet population was –2.1 (‒2.4 to ‒1.9) vs. –1.7 (‒1.9 to ‒1.5), respectively (Table 3). Significant (95% CIs of LSMDs do not include 0) reductions in the mean daily number of micturitions with vibegron compared with placebo were seen as early as week 4 for patients with OAB dry and at week 2 for patients with OAB wet and were maintained through week 8 (Figure 2; Supplementary Table 2). No significant differences in reductions (95% CIs of LSMD include 0) from baseline at week 12 in the mean daily number of micturitions were seen between tolterodine and placebo for the dry or wet populations in this study.

#### 3.2.3. Responder Analysis for Urgency Episodes

**Figure 3:**
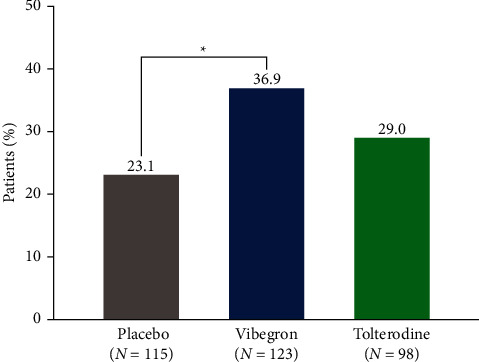
Percentage of patients with OAB dry achieving ≥50% reduction from baseline in mean daily urgency episodes. ^∗^*P* < 0.05 vs. placebo using mixed model for repeated measures.

A significantly greater percentage of patients with OAB dry treated with vibegron compared with placebo achieved ≥50% reduction from baseline at week 12 in urgency episodes (36.9% vs. 23.1%, respectively; nominal *P* < 0.05; Figure 3). No significant differences were observed from baseline at week 12 in urgency episodes between tolterodine and placebo (29.0% vs. 23.1%, respectively; nominal *P* > 0.05) for patients with OAB dry in this study.

## 4. Discussion

In this subgroup analysis of patients with OAB dry and wet from the phase 3 EMPOWUR trial, treatment with vibegron was associated with significant improvements from baseline at week 12 in mean daily number of urgency episodes and micturitions compared with placebo. No significant differences were observed between tolterodine and placebo for either outcome in either the OAB dry or wet populations in this study. Statistically significant reductions in urgency episodes and micturitions were observed with vibegron vs. placebo as rapidly as weeks 2 and 4, respectively, and were sustained throughout the 12-week treatment period. In addition, a significantly greater percentage of patients with OAB dry receiving vibegron compared to placebo achieved a clinically meaningful improvement (≥50% reduction) in urgency episodes per day at week 12. Similarly, in patients with OAB wet, treatment with vibegron was associated with statistically significant improvement compared with placebo from baseline in mean daily number of urgency episodes and micturitions beginning at week 2, which was maintained through week 12.

While UUI is a commonly studied symptom of OAB, a survey of >16,000 individuals showed that, among respondents reporting symptoms of OAB, frequency and urgency are the most commonly reported, occurring in 85% and 54% of respondents, respectively [2]. The urgent and immediate need to urinate can lead to substantial bother and at times require patients to alter their daily life to manage their symptoms [2, 23–25]. Furthermore, the degree of patient-reported bother of OAB symptoms has been shown to increase with increasing frequency of urgency [23], and symptoms of urinary urgency have been shown to be the strongest predictor of patient bother (greater than UUI, frequency, or nocturia) associated with OAB in both men and women (*P* *<* 0.01) [6, 23].

Although OAB dry affects two-thirds of patients with OAB [2, 4], most clinical trials have reported efficacy outcomes overall or in patients with OAB wet. Results from studies reporting by OAB subtype, however, often suggest that patients with OAB wet may experience greater improvement than those without incontinence [16, 17], highlighting the importance of assessing efficacy outcomes in patients with and without UUI. In our analyses, treatment with vibegron was associated with slightly greater reductions from baseline at week 12 in urgency episodes in the OAB wet population compared with the OAB dry population (‒3.0 vs. ‒2.6, respectively); however, responses with placebo varied between populations, with patients with OAB wet experiencing greater improvement than those with OAB dry (‒2.4 vs. ‒1.6). High placebo responses in OAB clinical trials have been well documented, and such responses may be due to factors outside of pharmacologic treatment such as use of voiding diaries and contextual factors of being part of a clinical trial [26].

While patients with OAB wet in the present analyses experienced slightly greater improvement than those with OAB dry, it is important to note that treatment with vibegron was associated with significant improvement vs. placebo in OAB symptoms of urgency and micturition frequency in patients with OAB dry, extending results from the overall population. These results contrast previous reports showing significantly greater efficacy in patients with OAB wet and show that vibegron is similarly efficacious among patients without or with UUI. As treatment guidelines do not differentiate between OAB subtypes [1], these results suggest that vibegron can be a valuable pharmacologic treatment for patients with OAB irrespective of subtype. Clinically meaningful decreases with vibegron in urinary urgency, arguably the most bothersome symptom of OAB, address a need in the treatment of OAB to effectively manage urgency episodes to improve patient outcomes.

A potential limitation of these post hoc analyses is the relatively high placebo response that varied between the OAB dry and wet populations, but this observation is consistent with past OAB studies. Furthermore, the sample size for the EMPOWUR trial was calculated based on the overall patient population, and the trial was therefore not powered to detect significant differences between treatments in either subgroup.

## 5. Conclusions

In this post hoc analysis of patients with OAB dry and OAB wet, treatment with once-daily vibegron 75 mg was associated with significant improvement vs. placebo in urgency episodes and micturitions in both populations. These results are consistent with the results from the EMPOWUR study showing that vibegron significantly improves symptoms of OAB. Patients with OAB dry or OAB wet receiving vibegron experienced similar symptomatic improvements of OAB, including urinary urgency and micturitions, indicating that vibegron is similarly efficacious for these endpoints in patients with or without UUI.

## Figures and Tables

**Table 1:** Demographics and baseline clinical characteristics of patients with OAB dry and wet from the EMPOWUR trial.

Characteristic	Dry population^†^			Wet population^‡^			Overall population (FAS^§^)		
	Placebo (*N* = 115)	Vibegron (*N* = 123)	Tolterodine (*N* = 98)	Placebo (*N* = 405)	Vibegron (*N* = 403)	Tolterodine (*N* = 319)	Placebo (*N* = 520)	Vibegron (*N* = 526)	Tolterodine (*N* = 417)
*Mean (SD) age, y*	59.5 (14.6)	59.4 (14.0)	60.7 (12.4)	60.0 (13.0)	61.2 (13.1)	59.5 (13.4)	59.9 (13.3)	60.8 (13.3)	59.8 (13.2)
≥65, *n* (%)	52 (45.2)	50 (40.7)	38 (38.8)	168 (41.5)	192 (47.6)	128 (40.1)	220 (42.3)	242 (46.0)	166 (39.8)
≥75, *n* (%)	13 (11.3)	16 (13.0)	11 (11.2)	44 (10.9)	59 (14.6)	36 (11.3)	57 (11.0)	75 (14.3)	47 (11.3)
*Gender, n (%)*									
Women	81 (70.4)	88 (71.5)	68 (69.4)	364 (89.9)	361 (89.6)	284 (89.0)	445 (85.6)	449 (85.4)	352 (84.4)
Men	34 (29.6)	35 (28.5)	30 (30.6)	41 (10.1)	42 (10.4)	35 (11.0)	75 (14.4)	77 (14.6)	65 (15.6)
*Race, n (%)*									
White	81 (70.4)	100 (81.3)	63 (64.3)	325 (80.2)	322 (79.9)	254 (79.6)	406 (78.1)	422 (80.2)	317 (76.0)
Black or African American	20 (17.4)	18 (14.6)	21 (21.4)	59 (14.6)	56 (13.9)	48 (15.0)	79 (15.2)	74 (14.1)	69 (16.5)
Asian	12 (10.4)	3 (2.4)	12 (12.2)	17 (4.2)	24 (6.0)	14 (4.4)	29 (5.6)	27 (5.1)	26 (6.2)
Others	2 (1.7)	2 (1.6)	2 (2.0)	4 (1.0)	1 (0.2)	3 (0.9)	6 (1.2)	3 (0.6)	5 (1.2)
*Region, n (%)*									
US	114 (99.1)	123 (100.0)	96 (98.0)	349 (86.2)	349 (86.6)	280 (87.8)	463 (89.0)	472 (89.7)	376 (90.2)
Non-US	1 (0.9)	0	2 (2.0)	56 (13.8)	54 (13.4)	39 (12.2)	57 (11.0)	54 (10.3)	41 (9.8)
Mean (SD) urgency episodes per day	8.6 (5.0)	8.6 (4.4)	8.4 (3.9)	8.0 (4.6)	8.0 (4.4)	7.8 (3.9)	8.1 (4.7)	8.1 (4.4)	7.9 (3.9)
Mean (SD) micturitions per day	11.9 (3.8)	11.3 (3.5)	11.6 (3.0)	11.7 (4.1)	11.3 (3.4)	11.5 (3.2)	11.8 (4.0)	11.3 (3.4)	11.5 (3.2)

FAS, full analysis set; OAB, overactive bladder. ^†^All randomized patients with OAB dry at study entry who took ≥1 dose of double-blind study treatment and had ≥1 evaluable change from baseline micturition measurement. ^‡^All randomized patients with OAB wet at study entry who took ≥1 dose of double-blind study treatment and had ≥1 evaluable change from baseline micturition measurement. ^§^All randomized patients who took ≥1 dose of double-blind study treatment and had ≥1 evaluable change from baseline micturition measurement.

**Table 2:** Change from baseline at week 12 in mean daily number of urgency episodes^†^.

	Dry population^‡^			Wet population^§^		
	Placebo (*N* = 115)	Vibegron (*N* = 123)	Tolterodine (*N* = 98)	Placebo (*N* = 405)	Vibegron (*N* = 403)	Tolterodine (*N* = 319)
*Change from baseline at week 12*						
*n*	103	109	92	372	383	286
LS mean (SE)	‒1.6 (0.34)	‒2.6 (0.33)	‒2.0 (0.36)	‒2.4 (0.17)	‒3.0 (0.17)	‒2.8 (0.19)
95% CI	‒2.2 to ‒0.9	‒3.2 to ‒2.0	‒2.7 to ‒1.3	‒2.7 to ‒2.0	‒3.3 to ‒2.6	‒3.2 to ‒2.4
*Active ‒ placebo*						
LSMD (SE)	—	‒1.0 (0.47)	‒0.4 (0.49)	—	‒0.6 (0.24)	‒0.4 (0.26)
95% CI	—	‒2.0 to ‒0.1	‒1.4 to 0.5	—	‒1.0 to ‒0.1	‒1.0 to 0.1

LS, least squares; LSMD, LS mean difference. ^†^Analyzed using a mixed model for repeated measures with covariates for study visit, baseline number of urgency episodes, and treatment by study visit interaction. ^‡^All randomized patients with OAB dry at study entry who took ≥1 dose of double-blind study treatment and had ≥1 evaluable change from baseline micturition measurement. ^§^All randomized patients with OAB wet at study entry who took ≥1 dose of double-blind study treatment and had ≥1 evaluable change from baseline urge urinary incontinence measurement.

**Table 3:** Change from baseline at week 12 in mean daily number of micturitions^†^.

	Dry population^‡^			Wet population^§^		
	Placebo (*N* = 115)	Vibegron (*N* = 123)	Tolterodine (*N* = 98)	Placebo (*N* = 405)	Vibegron (*N* = 403)	Tolterodine (*N* = 319)
*Change from baseline at week 12*						
*n*	103	109	92	372	383	286
LS mean (SE)	‒1.0 (0.26)	‒1.8 (0.25)	‒1.3 (0.28)	‒1.7 (0.12)	‒2.1 (0.12)	‒2.0 (0.13)
95% CI	‒1.5 to ‒0.5	‒2.3 to ‒1.3	‒1.9 to ‒0.8	‒1.9 to ‒1.5	‒2.4 to ‒1.9	‒2.2 to ‒1.7
*Active ‒ placebo*						
LSMD (SE)	‒	‒0.8 (0.36)	‒0.3 (0.38)	‒	‒0.5 (0.17)	‒0.3 (0.18)
95% CI	‒	‒1.5 to ‒0.1	‒1.1 to 0.4	‒	‒0.8 to ‒0.1	‒0.6 to 0.1

LS, least squares; LSMD, LS mean difference. ^†^Analyzed using a mixed model for repeated measures with covariates for study visit, baseline number of micturitions, and treatment by study visit interaction. ^‡^All randomized patients with OAB dry who took ≥1 dose of double-blind study treatment and had ≥1 evaluable change from baseline micturition measurement. ^§^All randomized patients with OAB wet who took ≥1 dose of double-blind study treatment and had ≥1 evaluable change from baseline urge urinary incontinence measurement.

## Data Availability

Requests for the data from Urovant Sciences (e-mail: medinfo@urovant.com) will be considered from qualified researchers on a case-by-case basis.

## Supplementary Materials

Supplementary Materials Supplementary Table 1. Change from baseline in mean daily number of urgency episodes. Supplementary Table 2. Change from baseline in mean daily number of micturitions. Supplementary Figure 1. Patient disposition.
