# Supplementary material for: Vibegron for the Treatment of Patients with Dry and Wet Overactive Bladder: A Subgroup Analysis from the EMPOWUR Trial
Source: Int J Clin Pract. 2022 Apr 13;2022:6475014. doi: 10.1155/2022/6475014 (PMC9159226; doi:10.1155/2022/6475014)

Supplementary Table 1. Change From Baseline in Mean Daily Number of Urgency Episodes[†]

	Dry Population[‡]			Wet Population[§]		
	**Placebo (N=115)**	**Vibegron (N=123)**	**Tolterodine (N=98)**	**Placebo (N=405)**	**Vibegron (N=403)**	**Tolterodine (N=319)**
Baseline						
n	115	123	98	405	403	319
Mean (SD)	8.6 (5.03)	8.6 (4.42)	8.4 (3.89)	8.0 (4.56)	8.0 (4.39)	7.8 (3.88)
Week 2						
n	108	118	96	391	390	310
LS mean (SE)	–0.8 (0.25)	–1.5 (0.24)	–1.4 (0.27)	–1.2 (0.12)	–1.7 (0.12)	–1.7 (0.14)
LSMD (SE)	–	–0.8 (0.35)	–0.6 (0.37)	–	–0.5 (0.17)	–0.6 (0.18)
95% CI	–	–1.5 to –0.1	–1.3 to 0.1	–	–0.8 to –0.2	–0.9 to –0.2
Week 4						
n	105	116	88	364	381	292
LS mean (SE)	–0.9 (0.27)	–1.8 (0.26)	–1.6 (0.29)	–1.4 (0.13)	–2.1 (0.13)	–2.1 (0.15)
LSMD (SE)	–	–0.9 (0.37)	–0.7 (0.40)	–	–0.7 (0.19)	–0.8 (0.20)
95% CI	–	–1.7 to –0.2	–1.5 to 0.1	–	–1.1 to –0.4	–1.2 to –0.4
Week 8						
n	106	115	95	380	389	295
LS mean (SE)	–1.2 (0.31)	–2.2 (0.30)	–2.2 (0.34)	–1.9 (0.16)	–2.6 (0.16)	–2.4 (0.18)
LSMD (SE)	–	–1.0 (0.44)	–1.0 (0.46)	–	–0.7 (0.22)	–0.5 (0.24)
95% CI	–	–1.9 to –0.2	–1.9 to –0.1	–	–1.2 to –0.3	–1.0 to –0.1

LS, least squares; LSMD, LS mean difference.

[†]Analyzed using a mixed model for repeated measures with covariates for study visit, baseline number of urgency episodes, and treatment by study visit interaction.

[‡]All randomized patients with OAB dry at study entry who took ≥1 dose of double-blind study treatment and had ≥1 evaluable change from baseline micturition measurement.

[§]All randomized patients with OAB wet at study entry who took ≥1 dose of double-blind study treatment and had ≥1 evaluable change from baseline urge urinary incontinence measurement.

Supplementary Table 2. Change From Baseline in Mean Daily Number of Micturitions[†]

	Dry Population[‡]			Wet Population[§]		
	**Placebo (N=115)**	**Vibegron (N=123)**	**Tolterodine (N=98)**	**Placebo (N=405)**	**Vibegron (N=403)**	**Tolterodine (N=319)**
Baseline						
n	115	123	98	405	403	319
Mean (SD)	11.9 (3.77)	11.3 (3.46)	11.6 (3.05)	11.7 (4.07)	11.3 (3.41)	11.5 (3.19)
Week 2						
n	108	118	96	391	390	310
LS mean (SE)	–0.5 (0.23)	–1.0 (0.22)	–0.9 (0.24)	–0.8 (0.09)	–1.3 (0.09)	–1.1 (0.11)
LSMD (SE)	–	–0.5 (0.31)	–0.4 (0.33)	–	–0.5 (0.13)	–0.3 (0.14)
95% CI	–	–1.1 to 0.1	–1.1 to 0.2	–	–0.7 to –0.2	–0.6 to 0.0
Week 4						
n	105	116	88	364	381	292
LS mean (SE)	–0.5 (0.22)	–1.2 (0.22)	–1.1 (0.24)	–1.0 (0.10)	–1.6 (0.10)	–1.4 (0.11)
LSMD (SE)	–	–0.7 (0.31)	–0.6 (0.33)	–	–0.6 (0.14)	–0.4 (0.15)
95% CI	–	–1.3 to 0.0	–1.2 to 0.1	–	–0.8 to –0.3	–0.7 to –0.1
Week 8						
n	106	115	95	380	389	295
LS mean (SE)	–0.9 (0.24)	–1.6 (0.23)	–1.4 (0.25)	–1.4 (0.11)	–1.9 (0.11)	–1.7 (0.13)
LSMD (SE)	–	–0.7 (0.33)	–0.5 (0.35)	–	–0.6 (0.16)	–0.4 (0.17)
95% CI	–	–1.4 to –0.1	–1.2 to 0.2	–	–0.9 to –0.2	–0.7 to 0.0

LS, least squares; LSMD, LS mean difference.

[†]Analyzed using a mixed model for repeated measures with covariates for study visit, baseline number of micturitions, and treatment by study visit interaction.

[‡]All randomized patients with OAB dry at study entry who took ≥1 dose of double-blind study treatment and had ≥1 evaluable change from baseline micturition measurement.

[§]All randomized patients with OAB wet at study entry who took ≥1 dose of double-blind study treatment and had ≥1 evaluable change from baseline urge urinary incontinence measurement.

Supplementary Figure 1. Patient Disposition. FAS, full analysis set; OAB, overactive bladder.

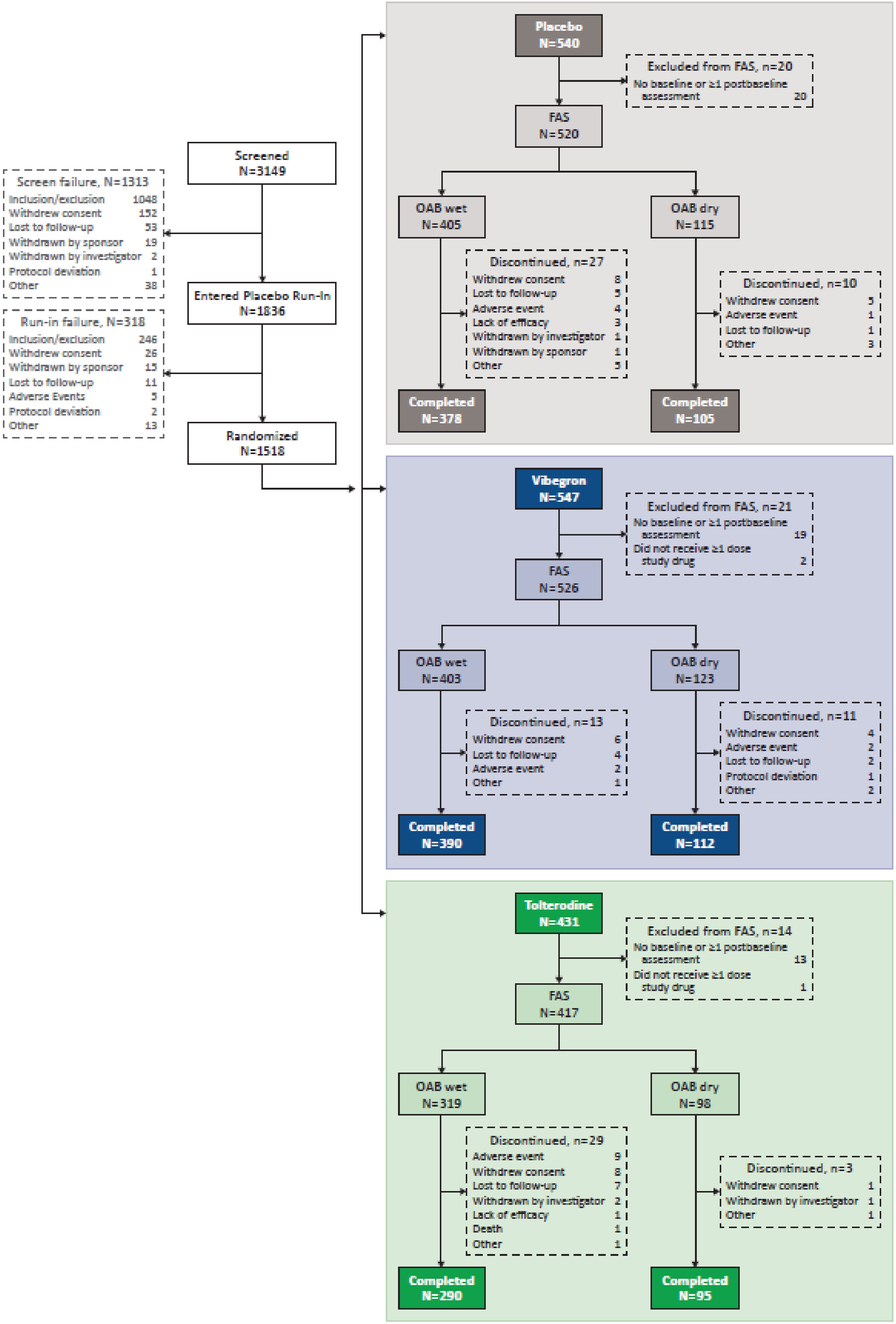

Supplement: Supplementary Materials — Supplementary Table 1. Change from baseline in mean daily number of urgency episodes. Supplementary Table 2. Change from baseline in mean daily number of micturitions. Supplementary Figure 1. Patient disposition. [file 6475014.f1.pdf]
